# Supplementary figures and images for: A Lipoxygenase 3 mutation reverses growth phenotypes in an Arabidopsis Plastid Lipase 3 overexpression line
Source: PLoS One. 2026 Jun 2;21(6):e0350738. doi: 10.1371/journal.pone.0350738 (PMC13229349; doi:10.1371/journal.pone.0350738)

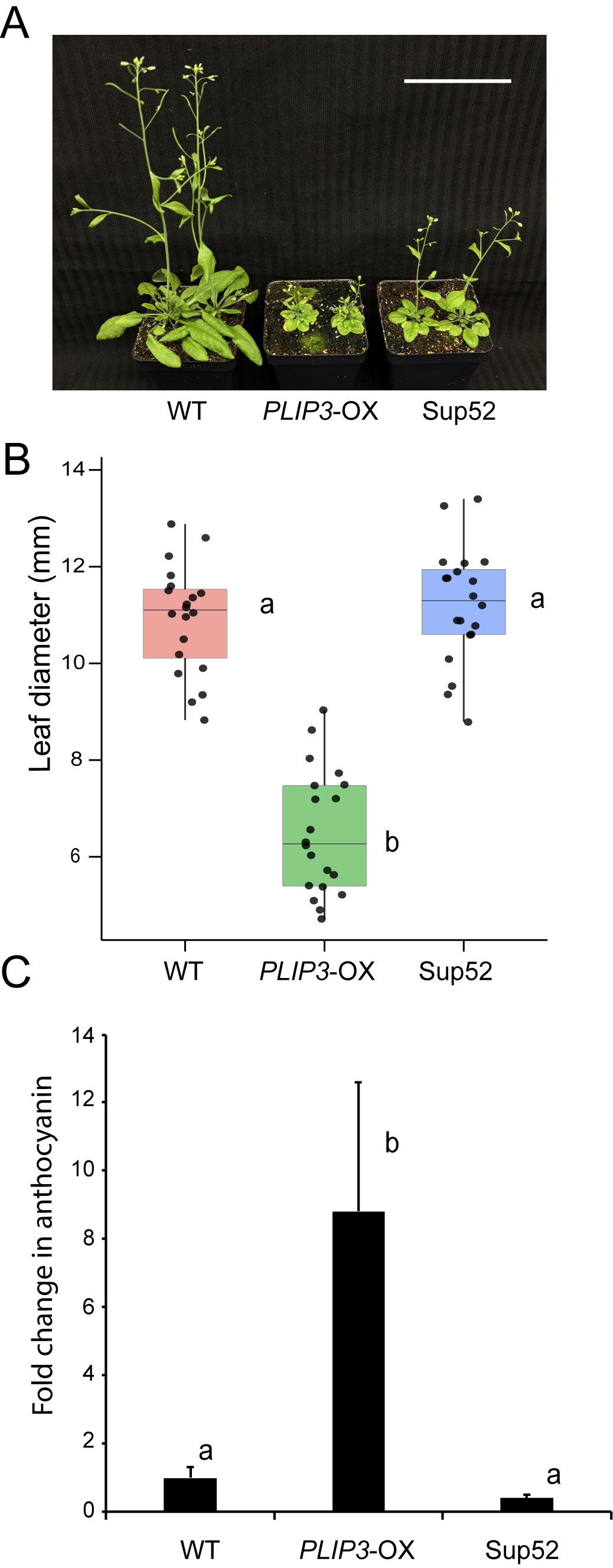

Supplement: S1 Fig — Shown are representative plants of wild type (WT), PLIP3-OX homozygous overexpression line (PLIP3-OX) and the #52 suppressor mutant line in the PLIP3-OX homozygous background (Sup52). The scale bar indicates 4 cm. (B) Leaf diameter of 6-week-old plants. N = 20, error bars indicate SD. Statistical analysis was performed in R using ANOVA followed by Tukey’s multiple comparison test to compare the leaf diameter in WT to that of PLIP3-OX and Sup52 plants. The different letters indicate a difference of the means with p < 0.05. (C) Anthocyanin levels in 4-week-old plants. N = 20, error bars indicate SD. Statistical analysis was performed in R using ANOVA followed by Tukey’s multiple comparison test to compare the leaf diameter in WT to that of PLIP3-OX and Sup52 plants. The different letters indicate a difference of the means with p < 0.05. Results were normalized to WT = 1. (TIF) [file pone.0350738.s001.tif]

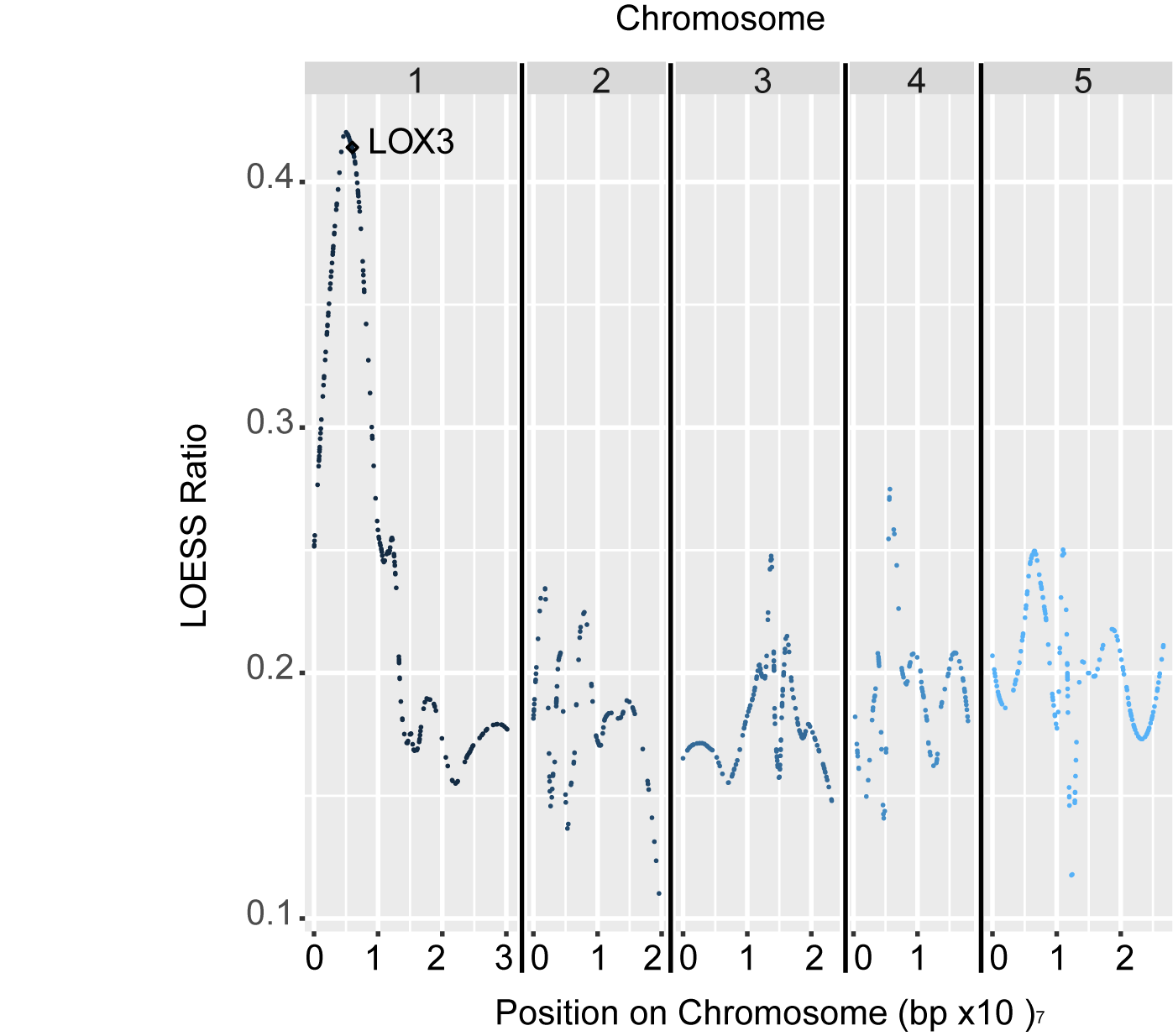

Supplement: S2 Fig — Shown is the chromosome location on the X-axis and the LOESS (locally estimated scatter plot smoothing) ratio variable as defined in [28], indicting the probability for the causal mutation for the selected phenotype. The opposition of the prime candidate LOX3 on chromosome 1 is indicated. (TIF) [file pone.0350738.s002.tif]

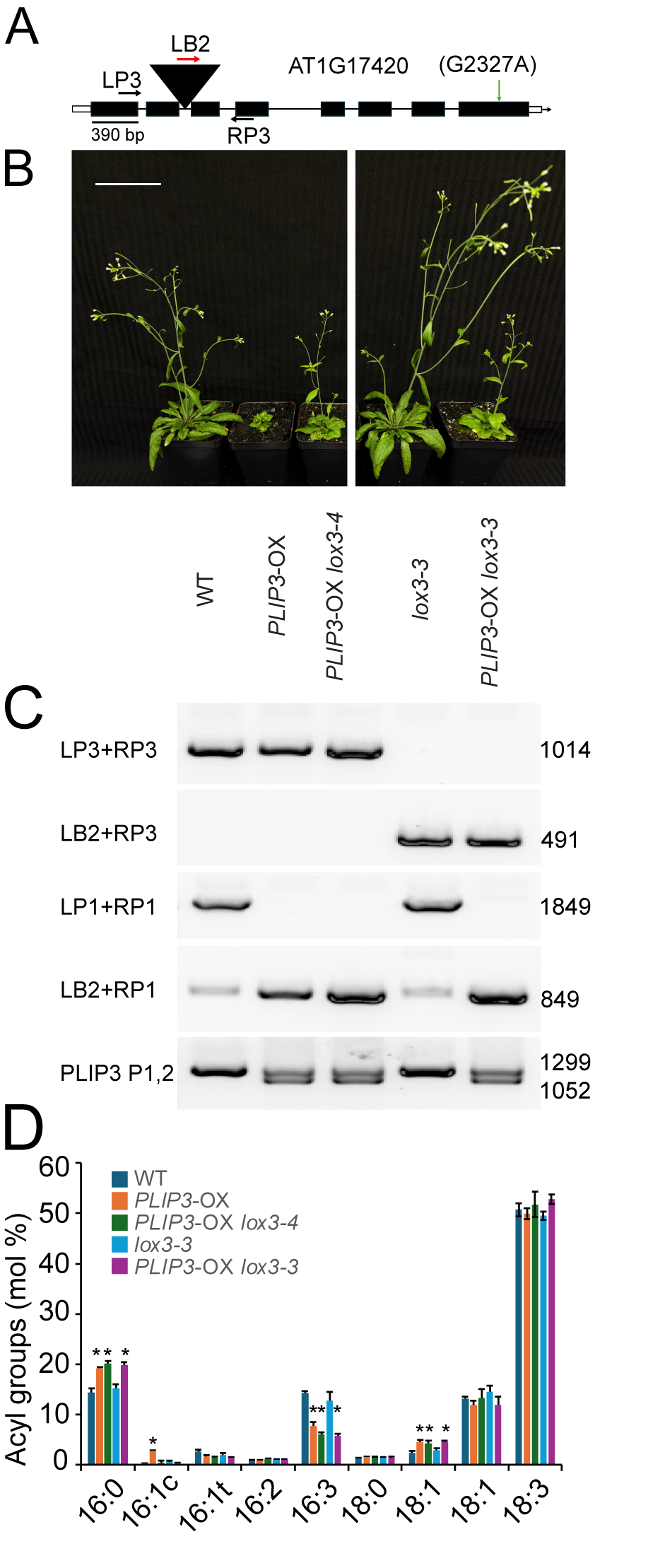

Supplement: S3 Fig — (A) Schematic drawing of the LOX3 locus (AT1G17420) indicating the location of the G2327A point mutation in the lox3−4 mutant allele. Black boxes represent exons, and black lines introns. The triangle indicates the T-DNA (not drawn to scale) in the lox3−3 mutant allele. A left border primer in the T-DNA is indicated in red (LB2, not drawn to scale). Primers flanking the T-DNA insertion site in lox3−3 (LP3, RP3) are indicated with black arrows (not drawn to scale). Open boxes indicate untranslated regions (only partly shown). (B) Phenotypes of six-week-old representative plants of WT, PLIP3-OX, PLIP3-OX lox3−4 (aka Sup52), lox3−3, and the PLIP3-OX; lox3−3 mutant (from left to right). The plants were homozygous at all indicted loci. The scale bar represents 4 cm. (C) Gel image of PCR genotyping results for the lines shown in (B) using primers as indicated in Fig 1B, the legend to Fig 2, and S3A Fig. The predicted fragment lengths are indicated (bp). (D) The bar graph shows total fatty acyl composition (mol%) of the lines as indicated. n = 3, Student’s t test was applied to compare plants with wild-type background, WT and lox3−1, to plants carrying the PLIP3 overexpression construct, PLIP3-OX, PLIP3-OX lox3−4, and PLIP3-OX lox3−1 (*P < 0.05); error bars show SD. (TIF) [file pone.0350738.s003.tif]

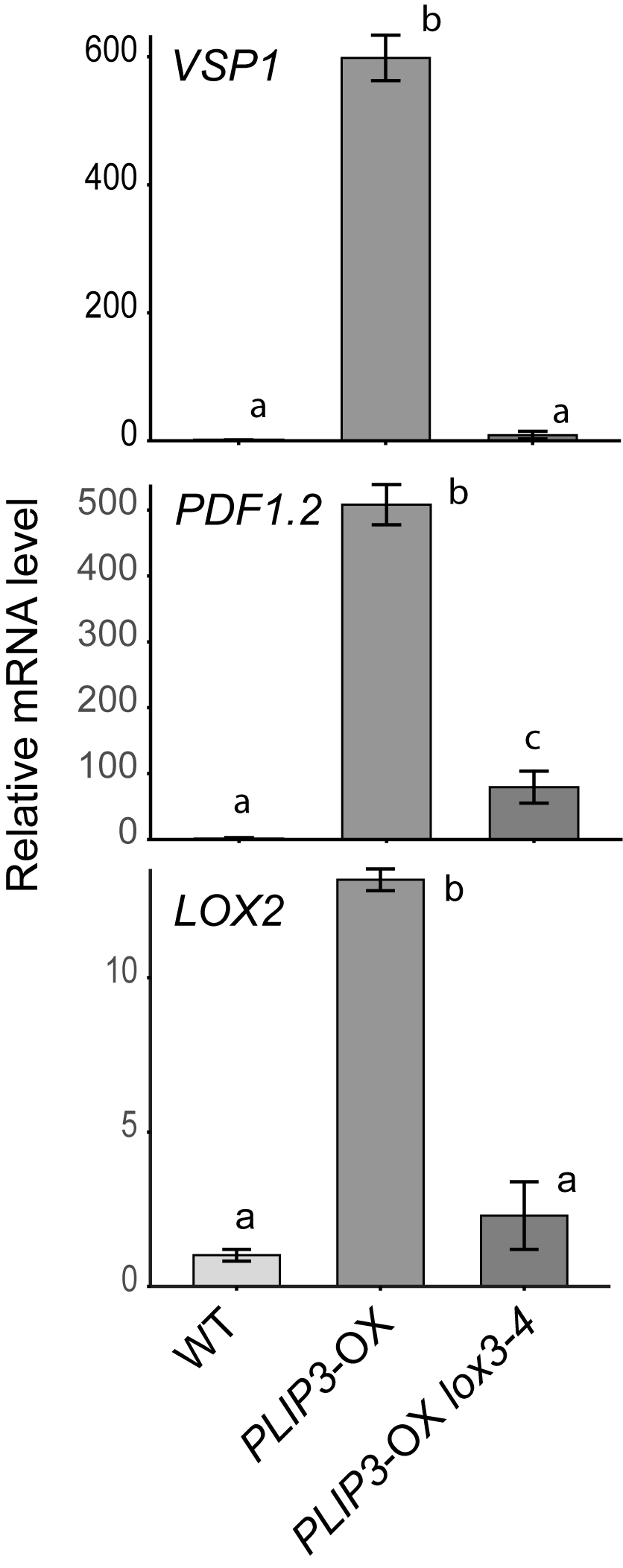

Supplement: S4 Fig — Real time quantitative PCR (RT-qPCR) was used to quantify the expression of the three JA-responsive genes VSP1 (At5g24780), PDF1.2 (At5g44420), and LOX2 (AT3G45140) in the three lines as indicated. Statistical analysis (n = 3) was performed in R using ANOVA followed by Tukey’s multiple comparison test to compare relative expression of the genes as indicated in WT, PLIP3-OX, and PLIP3-OX lox3–4 plants The different letters indicate a difference of the means with p < 0.05; error bars show SD. (TIF) [file pone.0350738.s004.tif]

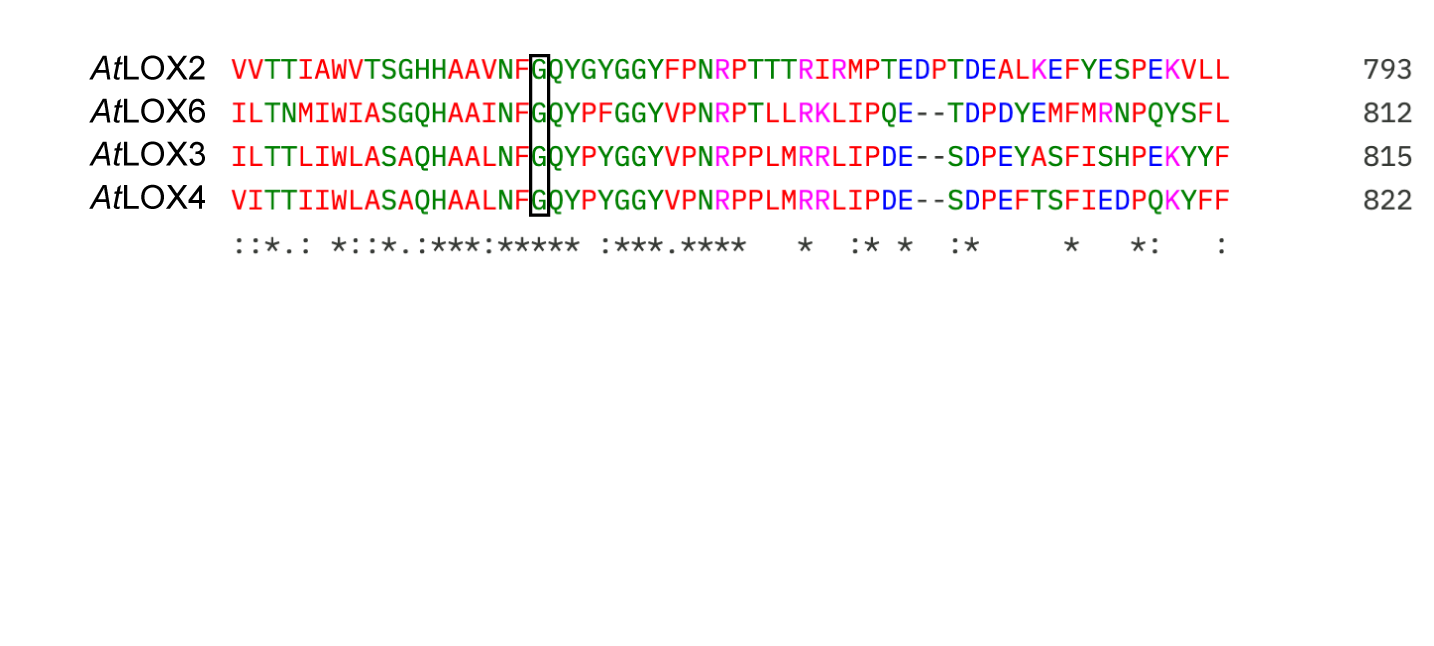

Supplement: S5 Fig — Alignment of a portion of the AtLOX3 protein with the corresponding sequences for AtLOX2 (AT3G45140), AtLOX4 (AT1G72520) and AtLOX6 (AT1G67560). The lox3–4 mutation affects a glycine residue that is conserved in the catalytic domain (black box). (TIF) [file pone.0350738.s005.tif]

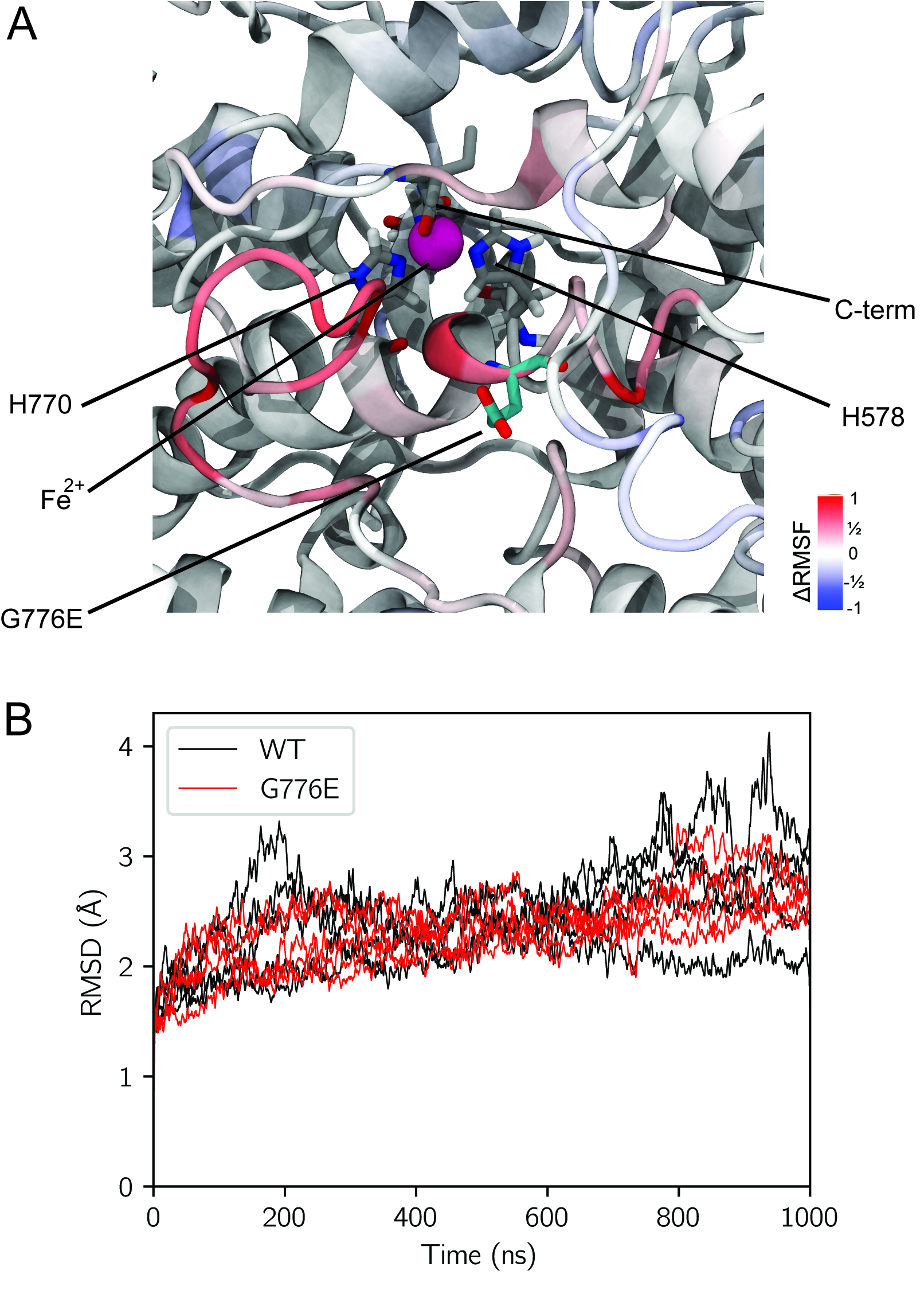

Supplement: S6 Fig — (A) Active site detail of the LOX3 structure shown in Figure 6C. The root mean-square fluctuation (RMSF) difference values for the WT and mutant lox3–4 structures show increased RMSF values for the G776E mutant protein near the C-terminus, which is close to the active site. The structure is drawn with higher variability locations in the mutant version which are colored red, while protein regions with lower variability in the mutant protein are colored blue. In addition to the labels in Figure 6C, the two active site histidines, H770 and H578, are pointed out in blue. They coordinate the iron (red sphere). (B) Molecular dynamics comparison of the structural integrity of the ATLOX3 WT and G776E mutant protein indicates that the protein structure remains intact. The root mean-square deviations for the two structures are shown for four simulations, each over 1000 ns. (TIF) [file pone.0350738.s006.tif]

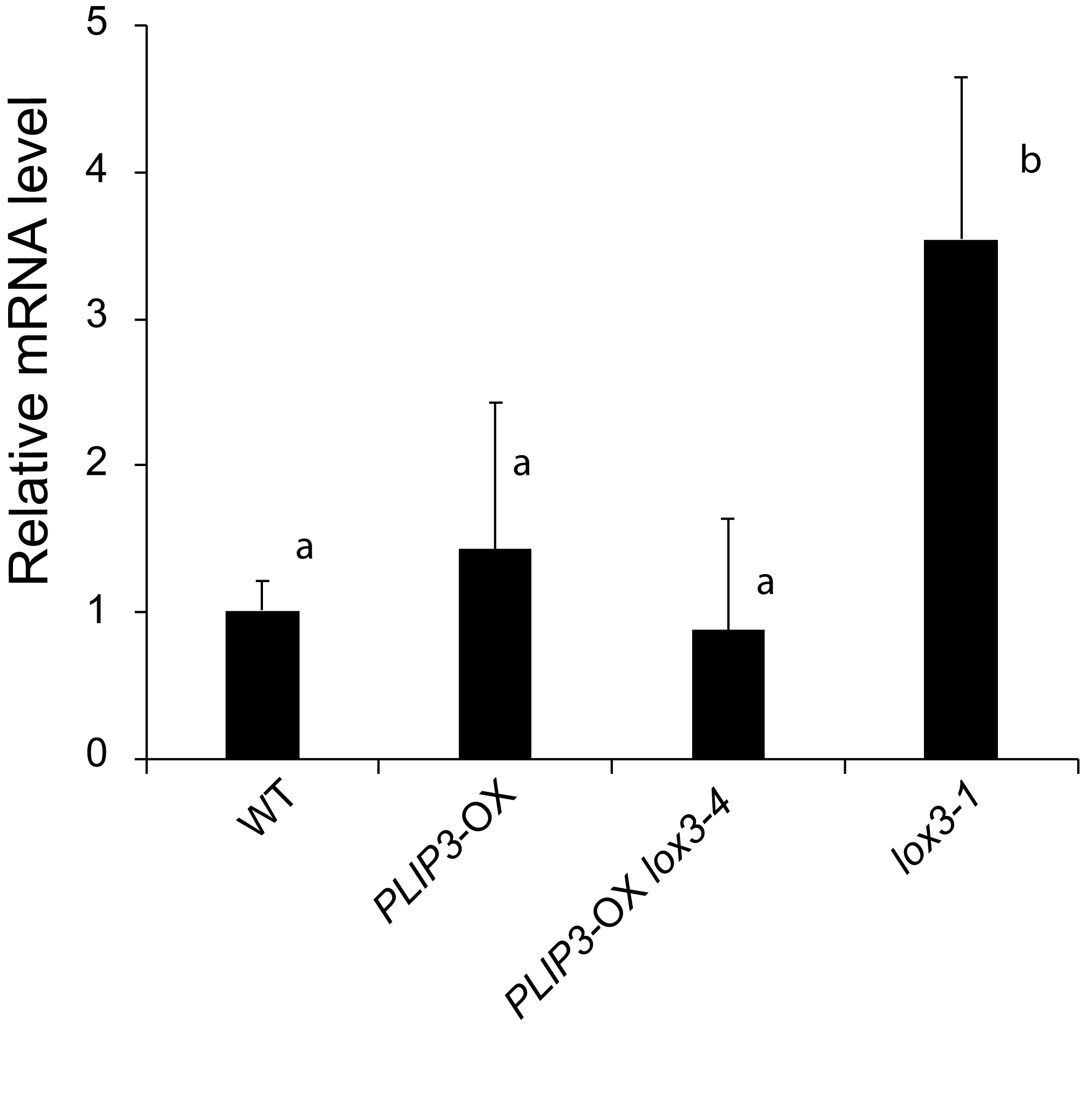

Supplement: S7 Fig — Statistical analysis was performed in R using ANOVA followed by Tukey’s multiple comparison test to compare relative expression in WT to that of PLIP3-OX, PLIP3-OX lox3−4, and lox3−1 plants. The different letters indicate a difference of the means with p < 0.05. (TIF) [file pone.0350738.s007.tif]
