# Supplementary material for: A Lipoxygenase 3 mutation reverses growth phenotypes in an Arabidopsis Plastid Lipase 3 overexpression line
Source: PLoS One. 2026 Jun 2;21(6):e0350738. doi: 10.1371/journal.pone.0350738 (PMC13229349; doi:10.1371/journal.pone.0350738)
Supplement: S1 Table — (DOCX) [file pone.0350738.s008.docx]

**S1 Table. Primers used in the study**

| Primer | Sequence 5’-3’ | Description |
| --- | --- | --- |
| PLIP3-P1 | TCCTGTTGAAGATGCTCAGA | PLIP3 genotyping forward primer |
| PLIP3-P2 | AGAGATAGAGACCACTTCCG | PLIP3 genotyping reverse primer |
| RP1 | CAGTCTGATAATCGCTACGCCTAG | See Fig. 2B |
| LP1 | GCAACTGCCTGGTATTATCACTCAAC | See Fig. 2B |
| LB1 | GTACCGGCAGGCTGAAGTCC | See Fig. 2B |
| RP2 | CACTCCGCTCTTACTGACGAC | See Fig. 3A |
| LP2 | GTTAGAATCGACACGAGGTCG | See Fig. 3A |
| LB2 | ATTTTGCCGATTTCGGAAC | See Fig 3A and Sup Fig 3A |
| RP3 | TATCAGCACCCAACTTGATCC | See Sup Fig. 3A |
| LP3 | CGCATTTCAAGAAGAGGACAC | See Sup Fig. 3A |
| VSP1 F | GGGCGTACTGGTCGTGGTTA | VSP1 qPCR Forward primer |
| VSP1 R | TCCCGAGTTCCAAGAGGTTTT | VSP1 qPCR Reverse primer |
| PDF1.2 F | TGTTCTCTTTGCTGCTTTCGACGC | PDF1.2 qPCR Forward primer |
| PDF1.2 R | TGTGTGCTGGGAAGACATAGTTGC | PDF1.2 qPCR Reverse primer |
| LOX2 F | GCCATTGAGTTGACTTGTCC | LOX2 qPCR Forward primer |
| LOX2 R | CACTTAGTTGTCTATTTGCCGC | LOX2 qPCR Reverse primer |
| UBQ10 F | TCCGGATCAGCAGAGGCTTA | UBQ10 qPCR Forward primer |
| UBQ10 R  HAK F  HAK R  LOX3 F  LOX3 R | TCAGAACTCTCCACCTCAAG  TAGCCTGTCCCGACCATCTC  AAGGCATGATCACAAGGAATCAGA  GCCGATCTAATTCGCAGAGGA  AAAAGACCGTCGTTGGCGTA | UBQ10 qPCR Reverse Primer  HAKAI qPCR Forward primer  HAKAI qPCR Reverse primer  LOX3 qPCR Forward primer  LOX3 qPCR Reverse primer |
