# Supplementary material for: A Lipoxygenase 3 mutation reverses growth phenotypes in an Arabidopsis Plastid Lipase 3 overexpression line
Source: PLoS One. 2026 Jun 2;21(6):e0350738. doi: 10.1371/journal.pone.0350738 (PMC13229349; doi:10.1371/journal.pone.0350738)
Supplement: Attachment — Submitted filename: Response Letter.docx [file pone.0350738.s010.docx]

**LOX3 PloS ONE Response to Reviews**

**Responses in Blue**

Journal Requirements:

When submitting your revision, we need you to address these additional requirements.

1. Please ensure that your manuscript meets PLOS ONE's style requirements, including those for file naming. The PLOS ONE style templates can be found at

<https://journals.plos.org/plosone/s/file?id=wjVg/PLOSOne_formatting_sample_main_body.pdf> and

[https://journals.plos.org/plosone/s/file?id=ba62/PLOSOne_formatting_sample_title_authors_affiliations.pdf](https://urldefense.com/v3/__https:/track.editorialmanager.com/CL0/https:*2F*2Fjournals.plos.org*2Fplosone*2Fs*2Ffile*3Fid=ba62*2FPLOSOne_formatting_sample_title_authors_affiliations.pdf/1/010f019cd69a0ca1-86daf255-eb17-49be-a6f3-687c952612f8-000000/vcC29cXznrYN3stY9sMhPEWOZysS_PvRrZTAwnSDtBY=253__;JSUlJSUlJQ!!HXCxUKc!w3JnijFfAx1r6zNkXSFaN6kYBUePD71FfZ_DkayPGGEKlKNBZBA2MHsKbGgKpxuGQp6a5m_kIfiFKQ4$).

Response: Done

2. Please note that PLOS One has specific guidelines on code sharing for submissions in which author-generated code underpins the findings in the manuscript. In these cases, we expect all author-generated code to be made available without restrictions upon publication of the work. Please review our guidelines at <https://journals.plos.org/plosone/s/materials-and-software-sharing#loc-sharing-code> and ensure that your code is shared in a way that follows best practice and facilitates reproducibility and reuse.

Response; Does not apply

3. Thank you for stating in your Funding Statement:

“This work was primarily supported by the National Science Foundation (MCB-2203474) and in part by the Office of Basic Energy Sciences of the United States Department of Energy (Grant DE-FG02-91ER20021) and by MSU AgBioResearch (MICL02632). Z.B.A. was supported by the High School Honors Science, Math and Engineering Program at Michigan State University. This work was supported in part through computational resources and services provided by the Institute for Cyber-Enabled Research at Michigan State University.”

Please provide an amended statement that declares *all* the funding or sources of support (whether external or internal to your organization) received during this study, as detailed online in our guide for authors at <http://journals.plos.org/plosone/s/submit-now>.  Please also include the statement “There was no additional external funding received for this study.” in your updated Funding Statement.

Please include your amended Funding Statement within your cover letter. We will change the online submission form on your behalf.

Response: Done

4. Thank you for stating the following financial disclosure:

“This work was primarily supported by the National Science Foundation (MCB-2203474) and in part by the Office of Basic Energy Sciences of the United States Department of Energy (Grant DE-FG02-91ER20021) and by MSU AgBioResearch (MICL02632). Z.B.A. was supported by the High School Honors Science, Math and Engineering Program at Michigan State University. This work was supported in part through computational resources and services provided by the Institute for Cyber-Enabled Research at Michigan State University.”

Please state what role the funders took in the study.  If the funders had no role, please state: "The funders had no role in study design, data collection and analysis, decision to publish, or preparation of the manuscript."

If this statement is not correct you must amend it as needed.

Please include this amended Role of Funder statement in your cover letter; we will change the online submission form on your behalf.

Response: Done

5. If the reviewer comments include a recommendation to cite specific previously published works, please review and evaluate these publications to determine whether they are relevant and should be cited. There is no requirement to cite these works unless the editor has indicated otherwise. 

Response: Does not apply

Note: HTML markup is below. Please do not edit.]
Reviewers' comments:

Reviewer's Responses to Questions

**Comments to the Author**

1. Is the manuscript technically sound, and do the data support the conclusions?

The manuscript must describe a technically sound piece of scientific research with data that supports the conclusions. Experiments must have been conducted rigorously, with appropriate controls, replication, and sample sizes. The conclusions must be drawn appropriately based on the data presented.

Reviewer #1: Yes

Reviewer #2: Yes

2. Has the statistical analysis been performed appropriately and rigorously?

Reviewer #1: Yes

Reviewer #2: Yes

3. Have the authors made all data underlying the findings in their manuscript fully available?

The [PLOS Data policy](https://urldefense.com/v3/__http:/track.editorialmanager.com/CL0/http:*2F*2Fwww.plosone.org*2Fstatic*2Fpolicies.action*23sharing/1/010f019cd69a0ca1-86daf255-eb17-49be-a6f3-687c952612f8-000000/mVxPAalw0FSpn57Hw3qs_Qlw2GCXk2o_JpSuqlRxe7E=253__;JSUlJSU!!HXCxUKc!w3JnijFfAx1r6zNkXSFaN6kYBUePD71FfZ_DkayPGGEKlKNBZBA2MHsKbGgKpxuGQp6a5m_kJJyKLS8$) requires authors to make all data underlying the findings described in their manuscript fully available without restriction, with rare exception (please refer to the Data Availability Statement in the manuscript PDF file). The data should be provided as part of the manuscript or its supporting information, or deposited to a public repository. For example, in addition to summary statistics, the data points behind means, medians and variance measures should be available. If there are restrictions on publicly sharing data—e.g. participant privacy or use of data from a third party—those must be specified.

Reviewer #1: Yes

Reviewer #2: Yes

4. Is the manuscript presented in an intelligible fashion and written in standard English?

PLOS ONE does not copyedit accepted manuscripts, so the language in submitted articles must be clear, correct, and unambiguous. Any typographical or grammatical errors should be corrected at revision, so please note any specific errors here.

Reviewer #1: Yes

Reviewer #2: Yes

5. Review Comments to the Author

Please use the space provided to explain your answers to the questions above. You may also include additional comments for the author, including concerns about dual publication, research ethics, or publication ethics. (Please upload your review as an attachment if it exceeds 20,000 characters)

Reviewer #1: The manuscript by Mugume et al. reports a mutation in LOX3 that attenuates the effects of PLIP3 overexpression on the plant growth and on jasmonic acid production in Arabidopsis thaliana. The manuscript is clearly and concisely written based on the results, and no major concerns were identified. I have only one comment as follows.

In lines 93–99, the authors state that, to query the entire pathway from the release of fatty acid precursors through the biosynthesis of JA and other oxylipins to their subsequent perception, transduction, and modification by other signaling pathways, they performed a suppressor screen in the PLIP3-OX transgenic line. However, I could not find a discussion of this question in the manuscript, particularly in light of the enzymatic characteristics of LOX3 and PLIP3 and their potential interactions.

Response, we added a sentence at this location in the introduction to better explain the expectation of the suppressor screen.

Wang et al. (2018) suggest that PLIP3 preferentially targets phosphatidylglycerol in vivo, but its overexpression substantially decreases the 16:3 fatty acid content (Fig. 1D), which is specific to MGDG. This observation may indicate that overexpressed PLIP3 releases 18:3 from the sn-1 position of MGDG, which could then be utilized by LOX3 and subsequently for OPDA production. Nonetheless, in chloroplasts, the major LOX enzyme LOX2, and presumably LOX4 and LOX6 as well, are also capable of 13S-lipoxygenation. It is therefore unclear why the loss of LOX3 strongly suppresses JA production in the PLIP3-OX background, given that LOX2 and other LOXs would be expected to metabolize the 18:3 fatty acids released by PLIP3. Are the fatty acids excised by PLIP3 not suitable substrates for these LOX enzymes? Please include a discussion on the genetic and possible biochemical interactions between LOX3 and PLIP3 in relation to the biosynthesis of JA and other oxylipins.

Response: We have no proof, but this could be explained by compartmentalization of these enzymes within the chloroplast providing LOX3 more direct access to the PLIP3 generated substrate. We added a sentence in the conclusion section, where we think this speculation is most appropriate.

Reviewer #2: This manuscript reports a suppressor screen to dissect the dwarf and abnormal growth phenotypes caused by PLIP3 overexpression (PLIP3-OX) and identifies a suppressor as a new LOX3 allele (lox3-4; G776E). The overall story is easy to follow, and the genetic case is strengthened by the fact that independent lox3 T-DNA alleles (lox3-1 and lox3-3) also partially suppress PLIP3-OX phenotypes. The hormone/oxylipin trends are generally consistent with the proposed interpretation.
A few issues should be addressed to strengthen the conclusions. First, the PLIP3-OX insertion maps near AT5G01160 (HAKAI). While the authors note this, the manuscript still largely assumes the phenotype reflects PLIP3 overexpression alone. It would help to show that HAKAI expression is not perturbed in PLIP3-OX (e.g., RT-qPCR), or, if available, to include data from an independent PLIP3 overexpression line. At a minimum, the limitations around insertion-site effects should be stated more explicitly.

Response: We measured the expression of the *HAKAI* by quantitative PCR as the insertion is in the 5’-untranslated region of the gene. The gene is expressed in all lines tested and its expression might even be increased in the PLIP3-OX line possibly due to a stimulation of expression by JA. We have included the expression data in Fig 2D and added a sentence.

Second, the discussion of the lox3-4 mutation would benefit from clearer weighting of evidence. The structural prediction/MD analysis is interesting and offers a plausible explanation for how G776E could compromise LOX3 function, but without direct biochemical support (LOX activity measurements, product profiling, or LOX3 protein abundance/stability), it remains speculative. In its current form, I would place the MD/structure analysis in the Supplementary Information and keep the main text to a brief, cautious statement (e.g., “consistent with reduced LOX3 function”), unless functional validation is added. Related to this, LOX3 transcript (and ideally protein) levels would help distinguish expression/stability effects from catalytic impairment, and a simple 13-LOX activity assay would provide a direct test. If that is not feasible, a more explicit quantitative comparison of JA/OPDA and related oxylipins across lox3-4 and established lox3 null/strong alleles would still strengthen the argument.

Response: We decided to keep the MD analysis in the main paper as we think it makes a valid contribution to the understanding of the *lox3-4* phenotype and the underlying biochemistry. We did try to produce properly folded LOX3 proteins in *E. coli* but were only able to obtain the protein in inclusion bodies and were unable to assay their activity. We measured the expression of the *LOX3* gene in the different lines and determined that its expression was not reduced. This is now shown in S7 Fig. Hence the loss of function of the *lox3-4* allele is likely due to the change in the enzyme activity*.* This is now discussed at the end of the MD section.

Third, given the EMS origin of lox3-4, it would be helpful to state more clearly how background mutations were handled (e.g., number of backcross generations and whether key phenotypes/hormone profiles were reproduced after backcrossing). The replication with T-DNA lox3 alleles supports causality, but a short clarification here would improve transparency.

Response: We believe the introduction of the two different lox3 alleles by crossing into *PLI3*-OX fully addresses this point and does not need further clarification. Any contributions by background mutations are excluded by this approach.

Finally, the statistics and reporting could be tightened. Several panels appear to use multiple pairwise t-tests; ANOVA with an appropriate post hoc test (or an explicit multiple-comparison correction) would be more appropriate. Please also report sample sizes unambiguously (avoid “n = 4 or 5” without specifying which datasets correspond to each n)

Response: We added ANOVA with posthoc analysis as suggested, when samples of different genetic backgrounds were analyzed as in Fig 4. We also clarified whether n=4 or 5 in the legend of Fig 4.

Minor points: The screen mentions multiple phenotypic features, but the figures emphasize petiole length; adding rosette diameter/biomass (or summarizing key metrics) would make “partial recovery” more convincing.

Response: We added this information in S1 Fig.

Growth conditions (age, photoperiod, light intensity) should be reported consistently across figures.

Response: Only the age of plants was variable, which is indicated in the figure legends. All other conditions were as stated in the Methods and are not repeated in each figure legend.

The anthocyanin-related observation based on the reddish phase in lipid extraction would be more convincing with a simple quantification.

Response: We added quantitative anthocyanin data in S1 Fig and a new method section.

Please also correct minor typos (e.g., “lipoxgenase” → “lipoxygenase”) and ensure consistent terminology throughout.

Response: Done

6. PLOS authors have the option to publish the peer review history of their article ([what does this mean?](https://urldefense.com/v3/__https:/track.editorialmanager.com/CL0/https:*2F*2Fjournals.plos.org*2Fplosone*2Fs*2Feditorial-and-peer-review-process*23loc-peer-review-history/1/010f019cd69a0ca1-86daf255-eb17-49be-a6f3-687c952612f8-000000/PwpB0gvJKEou5F9WIhR7BqpHdg72kiwdcDKHM47E6KE=253__;JSUlJSUl!!HXCxUKc!w3JnijFfAx1r6zNkXSFaN6kYBUePD71FfZ_DkayPGGEKlKNBZBA2MHsKbGgKpxuGQp6a5m_kbVFrHIY$)). If published, this will include your full peer review and any attached files.


If you choose “no”, your identity will remain anonymous but your review may still be made public.


**Do you want your identity to be public for this peer review?** For information about this choice, including consent withdrawal, please see our [Privacy Policy](https://urldefense.com/v3/__https:/track.editorialmanager.com/CL0/https:*2F*2Fwww.plos.org*2Fprivacy-policy/1/010f019cd69a0ca1-86daf255-eb17-49be-a6f3-687c952612f8-000000/NM7_lcesSxHzYO1uNZpWiJKrQoX8TSRFh6UPha1ZUXw=253__;JSUl!!HXCxUKc!w3JnijFfAx1r6zNkXSFaN6kYBUePD71FfZ_DkayPGGEKlKNBZBA2MHsKbGgKpxuGQp6a5m_kHJUBmBE$).

Reviewer #1: No

Reviewer #2: No

[NOTE: If reviewer comments were submitted as an attachment file, they will be attached to this email and accessible via the submission site. Please log into your account, locate the manuscript record, and check for the action link "View Attachments". If this link does not appear, there are no attachment files.]

To ensure your figures meet our technical requirements, please review our figure guidelines: <https://journals.plos.org/plosone/s/figures>

Response: Done

You may also use PLOS’s free figure tool, NAAS, to help you prepare publication quality figures: <https://journals.plos.org/plosone/s/figures#loc-tools-for-figure-preparation>.

NAAS will assess whether your figures meet our technical requirements by comparing each figure against our figure specifications.
